# Pressure gradient measurement in the coronary artery using phase contrast (PC)-MRI: initial patient results towards noninvasive quantification of fractional flow reserve

**DOI:** 10.1186/1532-429X-18-S1-P218

**Published:** 2016-01-27

**Authors:** Zixin Deng, Sang Eun Lee, Zhaoyang Fan, Christopher T Nguyen, Qi Yang, Xiaoming Bi, Byoung Wook Choi, Daniel S Berman, Hyuk-Jae Chang, Debiao Li

**Affiliations:** 1 Cedars Sinai Medical Center, Los Angeles, CA USA; 2 Bioengineering, University of California, Los Angeles, Los Angeles, CA USA; 3 R&D, Siemens Healthcare, Los Angeles, CA USA; 4 Radiology, Severance Hospital, Yonsei University College of Medicine, Seoul, Korea (the Republic of); 5 Cardiology, Severance Cardiovascular Hospital, Yonsei University College of Medicine, Seoul, Korea (the Republic of)

## Background

**Figure 1:**
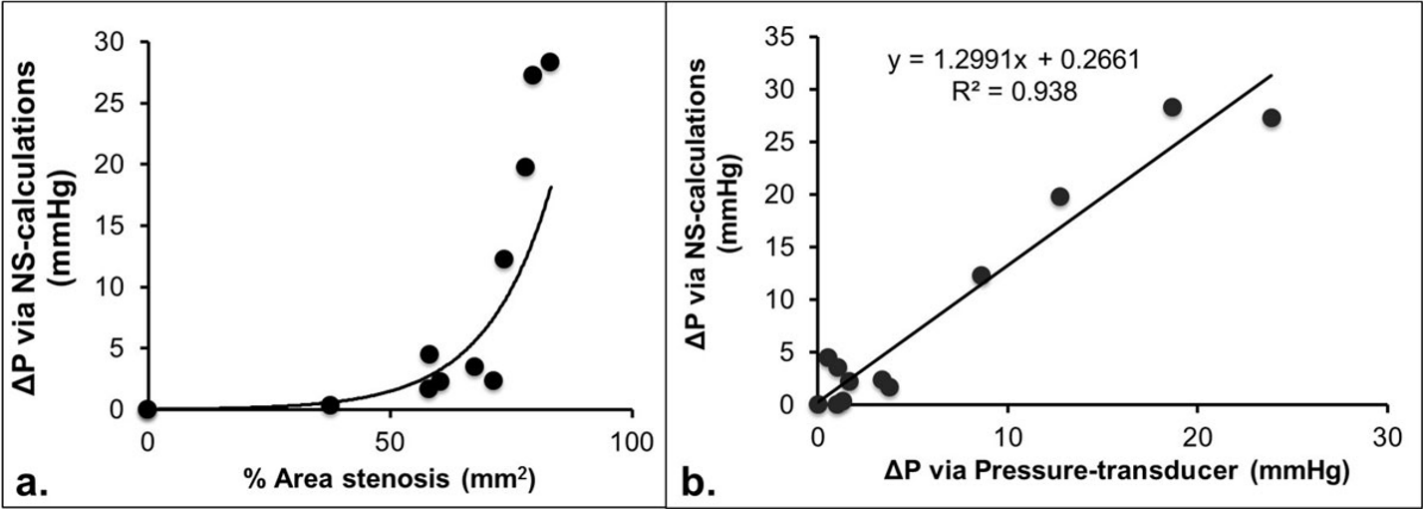
**a) % area stenosis versus PC-MRI derived ΔP measurement**. An exponential increase in ΔP was observed as % area stenosis increases. b) Pressure measurement comparison between ΔP calculated via NS-equations and Δ measured using pressure transducer. Excellent correlation (R^2^ = 0.938) was observed between the two techniques, validating the feasibility of PC-MRI derived ΔP.

Fractional flow reserve (FFR) is an invasive procedure evaluating the functional significance of an intermediate coronary stenosis in patients with coronary artery disease (CAD) [1]. Quantification of pressure gradient (ΔP) across a particular stenosis is the key to the determination of FFR. Noninvasive ΔP measurement (ΔP_MR_) using phase-contrast (PC)-MRI in conjunction with Navier-Stokes (NS) equations has been attempted in various vessels [2-4]. Our previous work has shown the feasibility of deriving ΔP_MR_ at various vessel diameters in a phantom (*fig.1a*) and excellent correlation between ΔP_MR_ and ΔP measured via a pressure-transducer (*fig.1b)*. This study aimed to investigate the feasibility of deriving ΔP_MR_ in healthy and diseased coronary arteries.

## Methods

Coronary PC-MRI acquisitions were ECG triggered (mid-diastole) and navigator gated (end-expiration) [5]. Fat-suppression pre-pulses were applied prior to the acquisitions to avoid chemical shift effects and increase vessel contrast [6]. Contiguous slices (4-9) were consecutively collected across the proximal coronary segment (healthy controls) or stenotic lesion (patients). Imaging parameters were: VENC=35-65 cm/s in all 3 directions, FA=15^o^, cardiac phase=2(~70 ms/phase), in-plane resolution = 0.5-0.6 × 0.5-0.6 mm^2^, slice thickness=3.2 mm and TA=2-4 min/slice at 3T. Eddy-current correction was done offline followed by NS calculations [7]. Protocol was performed on 11 healthy controls and 6 patients (one with known invasive FFR). Patient inclusion criteria: known/suspected CAD, ≥1 coronary lesion (proximal stenosis ≥30%) detected by CTA and/or invasive coronary angiography (ICA).

## Results

**Figure 2:**
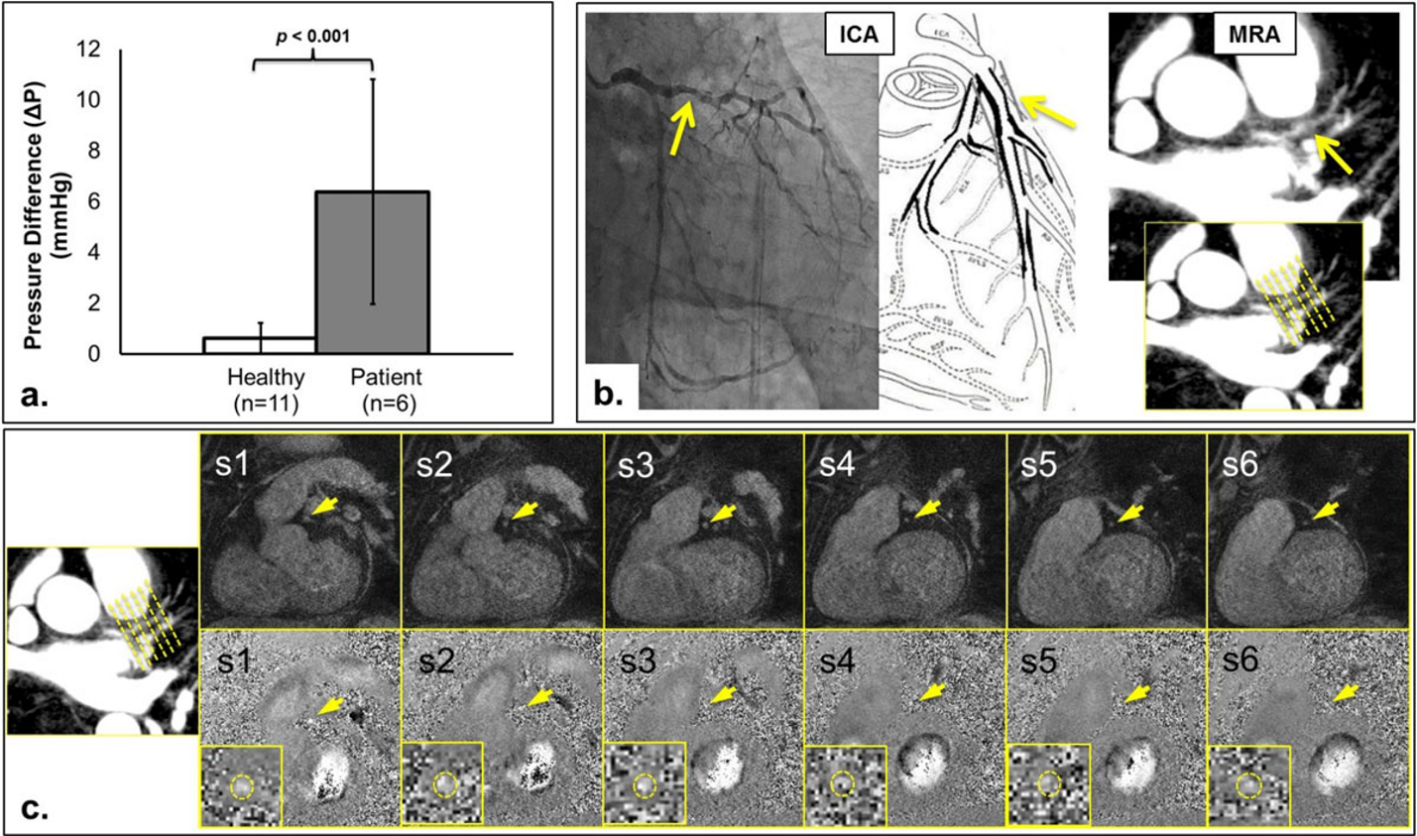
**a) The mean ± std ΔP value for health controls and patients were 0.6249 ± 0.4884 mmHg and 6.3960 ± 4.4302 mmHg, respectively**. The high standard deviation in the patient group is due to the range of the stenotic level among the patient group. b) Patient example (Patient A). Invasive coronary angiography (ICA) and magnetic resonance angiography (MRA). c) Patient A, six cross-sectional slices obtained from PC-MRI over the stenotic lesion at the proximal left anterior descending artery. *Top row*: flow compensated, *bottom row*: phase contrast (in the z-direction).

A significant (*p*<0.001) increase in ΔP_MR_ was seen in the patient group (6.40 ± 4.43 mmHg) vs. healthy controls (0.62 ± 0.49 mmHg) (*figre 2a*). CTA/ICA reports in 5/6 patients showed a range of stenoses of 30-50% (proximal left anterior descending coronary artery (*p*LAD)), but not significant enough to perform invasive FFR. ICA/FFR was performed in 1/6 patients (diffused, 50% lumen narrowing at *p*LAD, *fig 2b-c*) with FFR=0.56, suggesting a functionally significant lesion. The same patient showed a ΔP_MR_ of ~14 mmHg, likewise suggesting a functionally significant lesion (relatively high pressure drop).

## Conclusions

Preliminary results suggest that noninvasive quantification of ΔP_MR_ in both healthy and diseased coronary arteries is feasible. The patient with low FFR (high pressure drop), corroborating the ΔP_MR_ results, showed the feasibility of ΔP_MR_ in differentiating between a functionally significant and a non-significant lesion within the patient group. More patient studies with invasive FFR comparison are underway to further validate the approach. In addition, technical improvements in terms of spatial, temporal resolutions and reduction of noise are also being developed to further improve accuracy.
